# Subthreshold micropulse laser for retinal pigment epithelium detachment: subtype-stratified outcomes and predictors of treatment response

**DOI:** 10.3389/fmed.2026.1810012

**Published:** 2026-06-22

**Authors:** Wangting Li, Ting Xie, Jiafeng Ning, Zhi Li, Guoming Zhang, Xue Yao, Qingshan Chen

**Affiliations:** Shenzhen Eye Hospital, Shenzhen Eye Medical Center, Southern Medical University, Shenzhen, Guangdong, China

**Keywords:** macular disease, pigment epithelial detachment, retinal pigment epithelium, serous pigment epithelial detachment, subthreshold micropulse laser treatment

## Abstract

**Background:**

Pigment epithelium detachment (PED) arising from various macular diseases can significantly impair visual function. Although subthreshold micropulse laser treatment (SMLT) shows promise for PED, comparative data across subtypes and factors influencing outcomes remain limited.

**Methods:**

We included patients with visually significant macular PED who underwent SMLT between January 2017 and April 2023. Visual acuity and PED dimensions were recorded at baseline and follow-up. PEDs were classified as serous (sPED), drusenoid (dPED), or polypoidal (pPED) using fluorescein fundus angiography/indocyanine green angiography and optical coherence tomography. Within-group changes were assessed using paired *t*-tests (or Wilcoxon signed-rank tests where appropriate), and a linear regression analysis was used to identify prognostic factors.

**Results:**

Forty-two patients (42 eyes; mean age 60.90 years; 59.5% male patients) were included: 27 with sPED, 10 with dPED, and 5 with pPED. Patients with sPED were significantly younger (mean 53.00 years) than those with dPED (77.20) and those with pPED (71.00; *P* < 0.001). Overall, BCVA improved modestly after SMLT (mean −0.07 LogMAR; *P* = 0.046), although no individual subgroup reached significance. Significant PED area reduction was observed only in sPED (*P* = 0.043). Baseline BCVA was the strongest independent predictor of both BCVA improvement (β = −0.288, *P* = 0.001) and PED area reduction (β = −627,865, *P* = 0.004). Multiple SMLT sessions were often required for sPED, whereas 60.0% of pPED eyes needed adjunctive anti-VEGF therapy.

**Conclusion:**

SMLT was associated with modest visual and anatomic improvements, with statistically significant PED area reduction observed only in sPED eyes; the absence of a control arm limits causal inference. Baseline BCVA was the strongest predictor of outcomes. Larger controlled studies are needed to establish the efficacy of SMLT across PED subtypes.

## Background

Pigment epithelial detachment (PED) refers to the detachment of the retinal pigment epithelium (RPE) caused by fluid accumulation in the sub-RPE space due to systemic or ocular diseases, which often results in serious visual impairment (1, 2). PED can occur in various retinal diseases such as central serous chorioretinopathy (CSC), age-related macular degeneration (AMD), polypoidal choroidal vasculopathy (PCV), and drusen. It can also be secondary to certain ocular surgeries or systemic steroid use (3, 4). The pathogenesis of PED is complex, involving factors such as choroidal vascular abnormalities, RPE barrier damage, inflammatory responses, and mechanical traction. These pathological changes can lead to long-term visual decline, significantly impacting patients' quality of life (1, 2).

Although some patients with PED, such as those with CSC, may experience spontaneous resolution after the removal of systemic causative factors (16, 17), others may have persistent PED despite prolonged observation. With the progression of the disease, PED can induce choroidal neovascularization (CNV), exacerbating fluid and blood accumulation, disrupting retinal structure and function, and potentially causing retinal detachment (5, 6).

Recent studies have indicated that subthreshold micropulse laser treatment (SMLT) can promote functional recovery of the RPE layer and may be a more effective treatment for chronic, non-resolving patients with PED (7–9). By using a low-energy, subthreshold short-pulse laser, SMLT promotes RPE cell realignment and functional recovery, thereby reducing PED and improving vision. Compared with traditional laser photocoagulation, SMLT minimizes thermal damage to the RPE and surrounding retina, reducing potential negative impacts on vision (7–9). The high cost and scarcity of photosensitizers used in photodynamic therapy (PDT) have also propelled the adoption of SMLT as a viable treatment option for PED.

While SMLT has been most extensively studied in serous PED associated with CSC, where multiple studies have demonstrated favorable outcomes in promoting subretinal fluid resolution (7, 10), evidence regarding its efficacy in other PED subtypes remains limited. For drusenoid PED, only preliminary data suggest that 577-nm subthreshold micropulse laser may promote drusenoid PED regression in intermediate AMD (11); however, the sample sizes have been small and the follow-up periods have been short. For polypoidal PED associated with PCV, the standard of care remains anti-VEGF therapy, photodynamic therapy (PDT), or a combination of both (12, 13), and to our knowledge, no prior study has systematically evaluated SMLT as a treatment strategy for this subtype. This gap in the literature is clinically significant, as the high cost and limited availability of photosensitizers for PDT, particularly in China (14, 15), create an unmet need for alternative or adjunctive treatment approaches for polypoidal PED.

Despite the promising potential of SMLT for PED, comparative studies evaluating the differential responses of serous, drusenoid, and polypoidal PED subtypes to SMLT are lacking (4, 10, 11). In addition, systematic identification of baseline factors that predict treatment outcomes—such as patient age, baseline visual acuity, PED size and morphology, and the need for additional treatments—has not been performed. Understanding these factors is crucial for optimizing treatment protocols, enhancing therapeutic efficacy, and predicting patient response to treatment. This study aims to provide deeper insights into the clinical management of PED by analyzing the outcomes following SMLT and baseline factors associated with those outcomes across different PED subtypes.

## Methods

### Study design and patient selection

This study was a single-center, retrospective, observational case series aimed at evaluating outcomes following SMLT in patients with different types of PED and identifying baseline factors associated with those outcomes.

The co-primary endpoints of this study were (1) the change in best-corrected visual acuity (BCVA, LogMAR) from baseline to the 3-month follow-up as the functional outcome and (2) the change in PED area (μm^2^) from baseline to the 3-month follow-up as the anatomical outcome. A decrease in the LogMAR value indicates visual improvement; a negative change score (post–pre) therefore represents a gain in visual acuity, and a negative PED area change indicates lesion reduction.

### Clinical procedures and PED classification

All clinical procedures were performed by retinal disease specialists with more than 10 years of experience. Each patient underwent general information recording, ophthalmic examinations, SMLT, and any necessary additional treatments. Ophthalmic examinations consisted of fundus photography, fluorescein fundus angiography (FFA, performed with Spectralis HRA+OCT, Heidelberg,.Germany), indocyanine green angiography (ICGA, performed with Spectralis HRA+OCT, Heidelberg, Germany), the best-corrected visual acuity (BCVA) measurements, and OCT imaging (performed with Spectralis HRA+OCT, Heidelberg, Germany; Zeiss Cirrus HD-OCT 2000/5000, Zeiss, Germany) to acquire PED measurements (length, width, and area). FFA, ICGA, and OCT imaging were performed at baseline to determine vascular leakage, PED type, and PED measurements. FFA findings were classified as fluorescein staining, fluorescein leakage, and fluorescein pooling. ICGA findings were classified as vascular dilation, focal hyperfluorescence, and polypoidal lesions.

Based on the findings of FFA, ICGA, and OCT imaging, PEDs were classified into three subtypes: serous PED (sPED), drusenoid PED (dPED), and polypoidal PED (pPED). The fundus photograph of dPED presented as yellowish-white patchy lesions that can coalesce, showing a vague hyperfluorescence in the early phase of FFA, which gradually intensifies during the angiography process without leakage. OCT examination shows a medium-intensity reflective mass with the overlying pigment epithelial band appearing hyperreflective and undulating. The fundus photograph of sPED shows scattered serous PED lesions ranging from 0.5 PD to 3 PD in the posterior pole, with visible fluorescein filling on FFA. OCT reveals a smooth dome-shaped elevation with the lesion area filled with serous fluid, with a hyperreflective Bruch's membrane visible beneath it. The OCT features of pPED show finger-like projections, with the RPE layer and Bruch's membrane presenting a double-line sign. ICGA reveals polypoid lesions. In particular, only patients with pPED who were in the early stages or did not wish to undergo anti-VEGF treatment at the time were included in this study.

Additional treatments included repeated SMLT, routine anti-VEGF therapy, and anti-VEGF treatment *pro re nata* (PRN). Patients were followed up at 3 months, and ophthalmic examination, including BCVA and PED measurements, was performed.

### SMLT parameters

All patients received SMLT with a 577-nm yellow laser system (Supra Scan, Quantel Medical, Cedex, France). Laser spots of SMLT treatment were placed around the leakage points identified on the angiography. The SMLT protocol parameters were as follows: spot size of 140–160 μm and 200 ms exposure time with a 5% duty cycle (DC). A multi-spot mode without spacing between spots was chosen. Photocoagulation energy for each patient was titrated in the nasal upper quadrant of the retina using the mono-spot micropulse mode. The power titration was gradually increased until a visible burn was observed, and then, the power was reduced by 50% for individual treatment.

### Statistical analysis

Continuous variables were summarized as mean ± standard deviation (SD). Normality was assessed using Shapiro–Wilk tests. A one-way analysis of variance (ANOVA) was used to compare continuous variables among different PED types. Levene's test confirmed that the homogeneity of variance was satisfied for all baseline comparisons (all *P* > 0.036). For categorical variables, Fisher–Freeman–Halton exact tests (permutation-based) were used when expected cell counts were less than 5; otherwise, the chi-square tests were applied. Within-group pre- to posttreatment changes were assessed using paired *t*-tests; when normality was violated (PED area change), Wilcoxon signed-rank tests were also performed for confirmation. Univariate and multivariable linear regression analyses were performed to evaluate the associations between baseline characteristics and treatment outcomes. Effect sizes were reported as Cohen's *d* with 95% confidence intervals for within-group changes.

Because absolute PED area reduction is mathematically upper-bounded by baseline lesion size, the univariate associations between baseline PED dimensions and absolute area change were reexamined using proportional area change [(post–pre)/pre] as a sensitivity outcome, and an ANCOVA of posttreatment PED area on baseline PED area was performed to quantify the contribution of regression-to-the-mean effect (analogous to Supplementary Table S2 for the BCVA outcome).

Given the exploratory nature of the univariate analyses (nine tests per outcome), results should be interpreted with the understanding that some associations may represent chance findings; key findings were confirmed through the multivariable analysis. A sensitivity analysis excluding one patient with incomplete registration data was performed to assess robustness. A *p*-value of < 0.05 was considered statistically significant. All analyses were performed using Python 3.13 with scipy 1.17.1 and statsmodels 0.14.6.

## Results

### Study population and baseline characteristics

**Figure 1:**
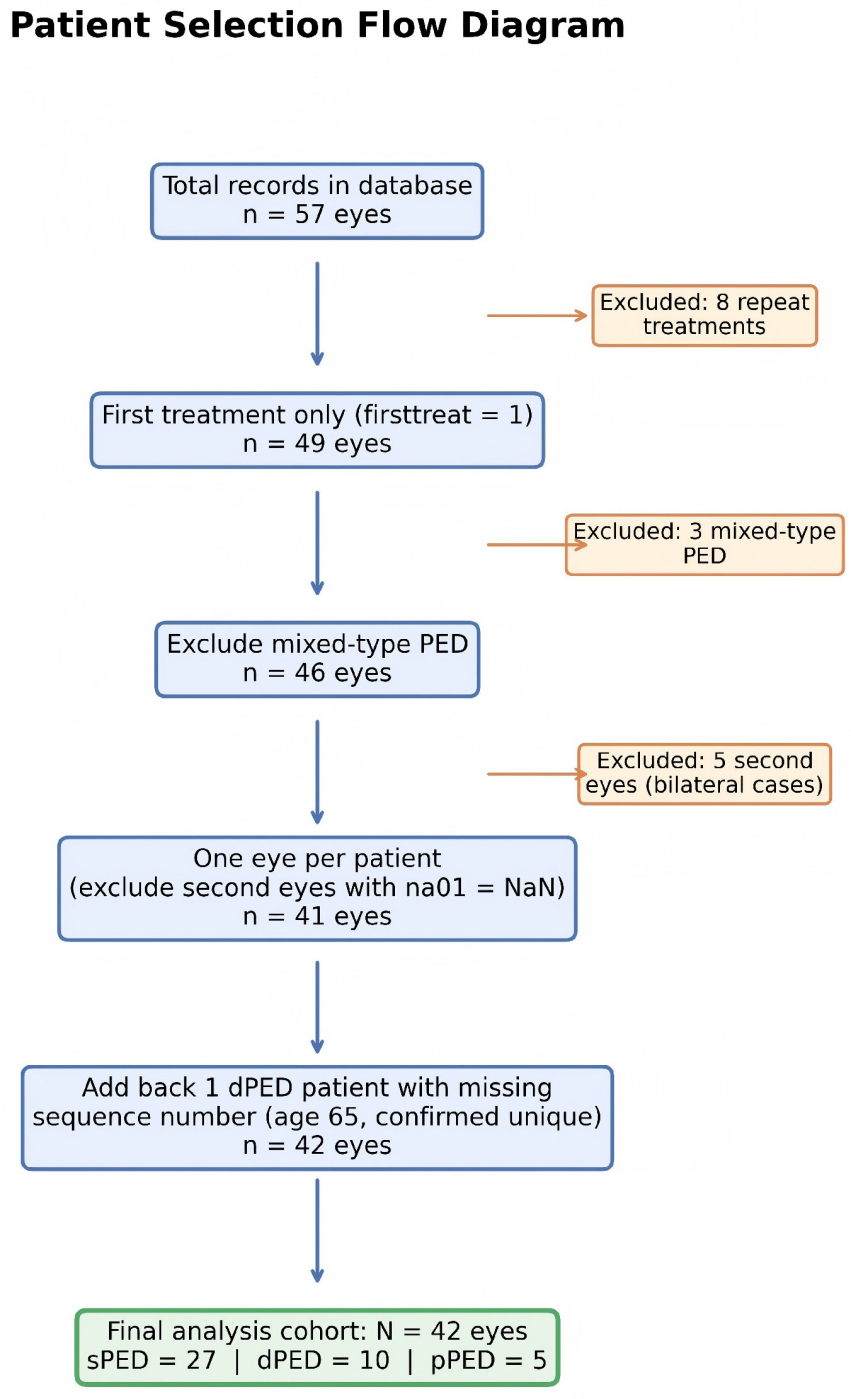
Flowchart of patient selection. Patients who underwent subthreshold micropulse laser treatment (SMLT) for pigment epithelium detachment (PED) at Shenzhen Eye Hospital between January 2017 and April 2023. Exclusion criteria were: secondary PED following retinal detachment surgery, mixed-type PED (concurrence of two or more PED subtypes), and second eyes of patients whose first affected eye was already enrolled. One drusenoid PED patient with incomplete registration data (no sequence number or patient identification number) was included following verification of data integrity; sensitivity analysis confirmed that exclusion of this patient did not materially affect any primary finding (*N* = 41 sensitivity result available in Supplementary Table S1). PED, pigment epithelium detachment; dPED, drusenoid pigment epithelium detachment; pPED, polypoidal pigment epithelium detachment; sPED, serous pigment epithelium detachment; SMLT, subthreshold micropulse laser treatment.

A total of 45 patients were initially enrolled. Three patients were excluded because of secondary PED due to retinal detachment surgery or mixed-type PED. Forty-two patients (42 eyes) were included in the final analysis (Figure 1). Among them, 27 eyes (64.3%) were classified as sPED, 10 eyes (23.8%) as dPED, and 5 eyes (11.9%) as pPED. The mean age of the study cohort was 60.90 years (SD 16.08 years; range, 36–84 years). Male patients comprised 59.5% of the cohort (25 of 42 patients).

Patient characteristics at baseline are summarized in Table 1. Patients with sPED were significantly younger than those with dPED or pPED [mean age, 53.00 (SD 13.73) vs. 77.20 (SD 7.42) vs. 71.00 (SD 9.49) years, respectively; *P* < 0.001]. The gender distribution differed significantly among PED types (*P* = 0.033, Fisher–Freeman–Halton exact test), with all five patients in the pPED group being male, compared with 63.0% (17 of 27) in the sPED group and 30.0% (3 of 10) in the dPED group. There was no significant difference in laterality of the affected eye among groups (*P* = 0.982).

**Table 1:** Baseline demographic and clinical characteristics of participants stratified by pigment epithelium detachment subtype.

Variable	Serous PED	Drusenoid PED	Polypoidal PED	Total	*P*
Age	53.00 (13.73)	77.20 (7.42)	71.00 (9.49)	60.90 (16.08)	< 0.001
Gender					
Male	17 (63.0%)	3 (30.0%)	5 (100.0%)	25 (59.5%)	0.033 (exact perm)^*^
Female	10 (37%)	7 (70.0%)	0 (0.0%)	17 (40.5%)	
Eye					
OD	17 (63.0%)	6 (60.0%)	3 (60.0%)	26 (61.9%)	0.982
OS	10 (37.0%)	4 (40.0%)	2 (40.0%)	14 (38.1%)	
Characteristics at baseline					
BCVA (logMAR)	0.43 (0.35)	0.50 (0.41)	0.37 (0.36)	0.44 (0.36)	0.787
PED width (μm)	1,338.93 (1,195.56)	1,282.30 (974.65)	2,007.75 (2,341.28)	1,390.37 (1,263.63)	0.597
PED length (μm)	319.30 (209.75)	285.30 (167.64)	312.50 (203.80)	310.34 (195.57)	0.900
Area of PED (μm^2^)	606,459.33 (1,020,528.03)	485,842.00 (521,749.78)	543,209.20 (790,820.03)	570,211.14 (885,386.89)	0.935
Laser parameters					
Laser power	408.22 (35.98)	420.00 (32.91)	405.00 (32.60)	410.64 (34.52)	0.617
Laser point	359.78 (222.74)	589.90 (275.67)	1,494.80 (1,830.46)	549.69 (712.81)	0.003†

Data are presented as mean (standard deviation, SD) for continuous variables and as number (percentage) for categorical variables. Continuous variables were compared among PED subtypes using one-way analysis of variance (ANOVA). For categorical variables, the Fisher-Freeman-Halton exact test (permutation-based, 100,000 resamples) was applied where expected cell counts were less than 5; otherwise, chi-square tests were used. Baseline PED dimensions (length, width, and area) were measured on optical coherence tomography (OCT) images at the initial visit. Laser parameters were recorded during the first SMLT session. BCVA, best-corrected visual acuity; LogMAR, logarithm of the minimum angle of resolution; mW, milliwatts; PED, pigment epithelium detachment; SD, standard deviation; SMLT, subthreshold micropulse laser treatment; μm, micrometers; μm^2^, square micrometers. ^*^P-value for gender was computed using Fisher–Freeman–Halton exact test (100,000 permutation resamples); all five patients with pPED were male. ^†^ANOVA for laser spots: P = 0.003. *Post hoc* comparisons were not performed, given the small and unequal subgroup sizes.

Baseline visual acuity and PED morphologic measurements did not differ significantly among the three PED types. The mean baseline BCVA was 0.43 (SD 0.35) LogMAR in the sPED group, 0.50 (SD 0.41) LogMAR in the dPED group, and 0.37 (SD 0.36) LogMAR in the pPED group (*P* = 0.787). Similarly, no significant between-group differences were observed in baseline PED length (*P* = 0.900), PED width (*P* = 0.597), or PED area (*P* = 0.935), although the wide standard deviations—particularly for PED area (mean 570,211 μm^2^; SD 885,387 μm^2^)—reflect substantial heterogeneity in lesion size within each group.

### SMLT treatment parameters

All patients received 577-nm subthreshold micropulse laser treatment with a mean energy of 410.64 mW (SD 34.52 mW), with no significant difference in power settings among groups (*P* = 0.617). However, the mean number of laser spots applied differed significantly across PED types (*P* = 0.003), reflecting the larger treatment areas required for more extensive lesions. Patients with pPED required a mean of 1,494.80 spots (SD 1,830.46), compared with 589.90 spots (SD 275.67) for dPED and 359.78 spots (SD 222.74) for sPED.

### Visual acuity outcomes

**Figure 2:**
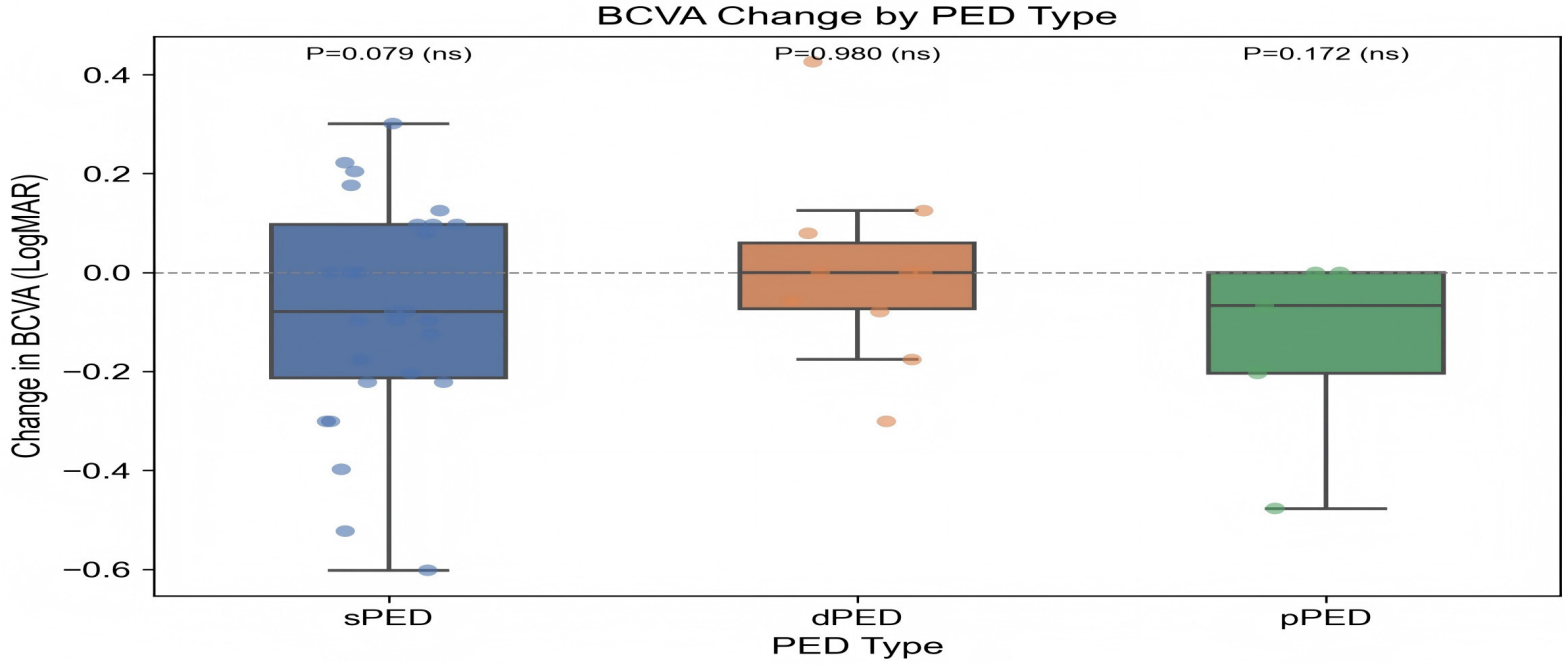
Improvement of BCVA at 3-month follow-up. Grouped box-and-whisker plot with individual data points (jittered) showing the distribution of BCVA change (post–pre, LogMAR) for each PED subtype. Boxes span the interquartile range (IQR; 25th−75th percentile); horizontal lines indicate the median; whiskers extend to 1.5 × IQR; individual observations beyond 1.5 × IQR are shown as open circles. A negative value on the *y*-axis denotes improvement in visual acuity. The horizontal dashed line at *y* = 0 indicates no change. *P*-values shown for each group are from two-tailed paired *t*-tests (within-group pre-to-post comparison). Effect sizes (Cohen's *d* with 95% CI) are annotated for each subgroup. The overall cohort change was −0.07 LogMAR (*P* = 0.046, paired *t*-test; *N* = 42), which is not displayed on this subgroup figure. BCVA, best-corrected visual acuity; CI, confidence interval; IQR, interquartile range; LogMAR, logarithm of the minimum angle of resolution.

At 3-month follow-up, the mean change in BCVA for the overall cohort was x0.07 LogMAR (SD 0.21), representing a modest improvement that reached statistical significance (*P* = 0.046, paired *t*-test; Table 2). However, when analyzed by PED type, no individual group demonstrated a statistically significant improvement in visual acuity at the α = 0.05 level. The sPED group showed a mean improvement of −0.08 LogMAR (SD 0.22; *P* = 0.079), corresponding to a small effect size (Cohen's *d* = −0.35; 95% CI, −0.89 to 0.19). The dPED group showed essentially no change in visual acuity (mean change, +0.00 LogMAR [SD, 0.19]; *P* = 0.980; Cohen's *d* = 0.01), and the pPED group showed a mean improvement of −0.15 LogMAR (SD 0.20; *P* = 0.172), corresponding to a medium effect size (Cohen's *d* = −0.74; 95% CI, −2.07 to 0.58), although this finding is limited by the small sample size (*n* = 5). There was no significant between-group difference in the magnitude of BCVA change (*P* = 0.406, ANOVA). Sensitivity analyses gave concordant non-significant between-group results (Welch's ANOVA *P* = 0.392; Kruskal–Wallis *P* = 0.507; permutation ANOVA *P* = 0.403, Figure 2).

**Table 2:** Comparative analysis of 3-month PED morphologic outcomes among different PED subtypes.

Classification	Serous PED	Drusenoid PED	Polypoidal PED	Total	*P*†
Characteristics at follow-up					
BCVA (logMAR)	0.35 (0.32)	0.50 (0.33)	0.22 (0.18)	0.37 (0.32)	0.232
PED width (μm)	217.70 (168.12)	267.50 (175.87)	288.45 (187.91)	317.24 (195.35)	0.582
PED length (μm)	985.86 (991.20)	1,185.20 (931.86)	1,979.85 (2,094.70)	1,463.10 (1,248.93)	0.214
Area of PED	350,381.72 (518,889.06)	436,647.00 (478,499.80)	470,991.41 (671,403.68)	385,279.37 (517,031.47)	0.842
Improvement of BCVA and absorption of PED					
BCVA (logMAR)‡	−0.08 (0.22)	0.00 (0.19)	−0.15 (0.20)	−0.07 (0.21)	0.406
BCVA change *P* (within)	0.079	0.980	0.172	NA	NA
PED area change (μm^2^)	−256,077.62 (624,951.69)	−49,195.00 (113,435.03)	−72,217.79 (134,350.16)	−184,931.78 (511,504.74)	0.491
PED area change *P* (within)	0.043^*^	0.203	0.296	NA	NA
Requirement of further treatment					
Treat with vegf					
N	26 (96.3%)	10 (100.0%)	2 (40.0%)	38 (90.5%)	0.002
Y	1 (3.7%)	0	3 (60.0%)	4 (9.5%)	
Treat with prn					
N	27 (100.0%)	10 (100.0%)	2 (40.0%)	39 (92.9%)	< 0.001
Y	0 (0.0%)	0 (0.0%)	3 (60.0%)	3 (7.1%)	
Treat with another under threshold laser					
N	21 (77.8%)	9 (90.0%)	5 (100.0%)	35 (83.3%)	0.574
Y	6 (22.2%)	1 (10.0%)	0 (0.0%)	7 (16.7%)	
Total	27	10	5	42	

Within-group pre-to-posttreatment changes were assessed using two-tailed paired t-tests. For the PED area change in the sPED group, where Shapiro–Wilk testing confirmed violation of normality (W = 0.648, P < 0.001), a Wilcoxon signed-rank test was also performed as a confirmatory non-parametric analysis. Between-group differences in change scores were evaluated using one-way ANOVA. Effect sizes for within-group changes are reported as Cohen's d with 95% confidence intervals (CI), computed as the mean change divided by the SD of the change. A negative BCVA change (LogMAR) indicates improvement in visual acuity. A negative PED area change indicates a reduction in lesion size. Because subgroup sizes were small and imbalanced (sPED n = 27; dPED n = 10; pPED n = 5) and PED area change deviated from normality, between-group comparisons of change scores were additionally evaluated using Welch's ANOVA (which does not assume equal variances), Kruskal–Wallis tests, and a permutation ANOVA (10,000 resamples). Homogeneity of variance was assessed using Levene's test. For the multivariable model of PED area change (Model C), robust inference was additionally obtained using HC3 heteroscedasticity-consistent standard errors and BCa bootstrap 95% confidence intervals (5,000 resamples), and a sensitivity analysis was conducted with the most influential observation (Cook's D = 0.9304) excluded. PRN, pro re nata; VEGF, vascular endothelial growth factor. ^*^Within-group P-values derived from paired t-tests (two-tailed). For the sPED PED area, the Wilcoxon signed-rank test P-value is additionally reported (P = 0.015), confirming the parametric result despite non-normal distribution. ^†^Between-group P-values derived from one-way ANOVA for continuous outcomes and from permutation-based Fisher exact tests for binary treatment variables. ^‡^Effect sizes (Cohen's d) for BCVA improvement: sPED: d = −0.35 (95% CI, −0.89 to 0.19); dPED: d = 0.01 (95% CI, −0.87 to 0.88); pPED: d = −0.74 (95% CI, −2.07 to 0.58). Wide confidence intervals for dPED and pPED reflect limited statistical power due to small subgroup sizes.

### PED morphologic outcomes

**Figure 3:**
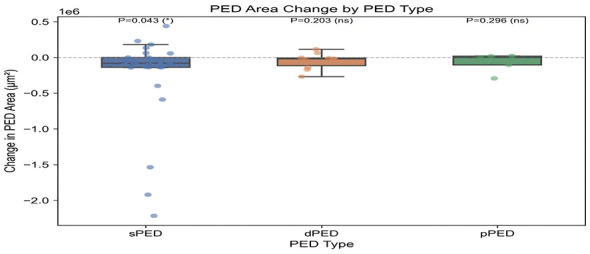
Change in PED area at 3-month follow-up. Grouped box-and-whisker plot with individual data points showing the distribution of PED area change (post–pre, μm^2^) for each PED subtype. Plot elements are as described for Figure 2. A negative value on the *y*-axis denotes a reduction in PED area. *P*-values are from paired *t*-tests; for sPED, the Wilcoxon signed-rank test *P*-value is additionally shown in parentheses (*P* = 0.015), which confirms the parametric result given the non-normal distribution of PED area change in this subgroup (Shapiro–Wilk *W* = 0.648, *P* < 0.001). The large variance within the sPED group reflects substantial interpatient heterogeneity in anatomic treatment response. Note: *y*-axis values have been truncated at −2,000,000 μm^2^ for clarity; one sPED outlier with an extreme PED area reduction is not fully displayed.

The sPED group demonstrated a mean reduction in the PED area of 256,078 μm^2^ (SD 624,952 μm^2^), which was statistically significant on both the paired *t*-test (*P* = 0.043) and the Wilcoxon signed-rank test (*P* = 0.015; Table 2). In contrast, neither the dPED group (mean reduction, 49,195 μm^2^ [SD 113,435 μm^2^]; *P* = 0.203) nor the pPED group (mean reduction, 72,218 μm^2^ [SD 134,350 μm^2^]; *P* = 0.296) showed statistically significant reductions in PED area. The between-group difference in PED area change was not statistically significant (*P* = 0.491, ANOVA). The large standard deviations relative to mean changes, particularly in the sPED group, suggest considerable interpatient variability in the anatomic response to SMLT. Sensitivity analyses revealed concordant non-significant between-group results (Welch's ANOVA *P* = 0.302; Kruskal–Wallis *P* = 0.722; permutation ANOVA *P* = 0.517, Figure 3).

### Additional treatment requirements

The need for adjunctive therapy differed markedly among PED subtypes (Table 2). Anti-VEGF therapy was significantly more common in the pPED group, with 60.0% (3 of 5) of patients receiving anti-VEGF injections, compared with 3.7% (1 of 27) of patients with sPED and 0.0% (0 of 10) of patients with dPED (*P* = 0.002, Fisher's exact test). PRN anti-VEGF treatment was administered exclusively to patients with pPED (60.0%, 3 of 5; *P* < 0.001). Repeat SMLT was performed for 22.2% (6 of 27) of sPED eyes, 10.0% (1 of 10) of dPED eyes, and none of the pPED eyes (*P* = 0.574).

### Factors associated with visual acuity improvement

**Figure 4:**
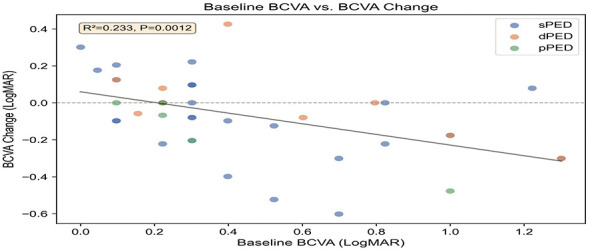
Association between baseline BCVA and the improvement at 3-month follow-up. Scatter plot of baseline BCVA (x-axis, LogMAR) against BCVA change at 3 months (*y*-axis, LogMAR; post–pre) for all 42 patients. Data points are color-coded and shaped by PED subtype: closed circles = sPED; open triangles = dPED; open squares = pPED. The regression line (solid) with 95% confidence band (shaded) is derived from the univariate linear regression model (β = −0.288; 95% CI, −0.455 to −0.121; *P* = 0.001; *R*^2^ = 0.233). A negative BCVA change indicates improvement. The negative slope reflects that patients with worse baseline acuity (higher LogMAR) tend to demonstrate greater improvement, consistent with a regression-to-the-mean effect (ANCOVA-estimated contribution: approximately 29%; see Supplementary Table S2). The horizontal dashed reference line is at *y* = 0 (no change).

Univariate linear regression analyses were performed to identify baseline factors associated with changes in BCVA across all 42 patients (Table 3). Baseline BCVA was the only variable significantly associated with the magnitude of BCVA improvement: each 1.0-LogMAR-unit increase in baseline acuity was associated with an additional 0.29-LogMAR-unit improvement (β = −0.288; 95% CI, −0.455 to −0.121; *P* = 0.001; *R*^2^ = 0.233, Figure 4). This association should be interpreted with caution, as approximately 29% of the effect may be attributable to regression-to-the-mean (ANCOVA β = 0.71, significantly less than 1.0; *P* = 0.001). Age (β = 0.002; *P* = 0.352), gender (β = 0.042; *P* = 0.305), baseline PED dimensions (all *P* > 0.25), number of laser spots (*P* = 0.721), and ICGA classification (*P* > 0.63 for all categories) were not significantly associated with BCVA change.

**Table 3:** Univariate linear regression analysis of the improvement of BCVA and PED with the baseline characteristics of participants.

Variables	Change of BCVA (logMAR)		Change of area of PED	
	*P*	β (95%CI)	*P*	β (95%CI)
Characteristics at baseline				
Age	0.352	0.00 (0.00, 0.00)	0.502	3,388.05 (−6717.22, 13,493.31)
Gender (Female)	0.305	0.05 (−0.08, 0.19)	0.596	−86,641.73 (−414,496.23, 241,212.77)
BCVA (logMAR)	0.001^*^	−0.29 (−0.46, −0.12)	0.004†	−627,864.55 (−1,036, 825.11, −218,903.99)
Performance of ICGA				
ICGA polypoidal lesions	0.822	−0.01 (−0.11,0.09)	0.564	−68,140.6 (−304889.19, 168,607.79)
ICGA focal hyperfluorescence	0.636	−0.02 (−0.12,0.07)	0.479	−81,273.5225 (−311,351.70, 148,804.66)
Performance of PED				
Baseline PED length	0.274	0.00 (−0.00, 0.00)	0.000†	−1,895.45 (−2,492.35,−1,298.55)
Baseline PED width	0.254	0.00 (−0.00, 0.00)	0.000†	−255.24 (−358.81,−151.67)
Baseline PED area	0.471	0.00 (0.00, 0.00)	0.000†	−0.50 (−0.59,−0.40)
Laser parameters				
Laser point	0.721	0.00 (−0.00, 0.00)	0.968	4.58 (−224.73, 233.88)

A negative β coefficient indicates that higher values of the predictor are associated with greater improvement (more negative LogMAR change) in BCVA/greater reduction in PED area. 95%CI: 95% Confidence interval. R^2^ = coefficient of determination. ^*^Bonferroni-corrected threshold for nine simultaneous tests: α = 0.0056. Baseline BCVA (P = 0.001) survives the uncorrected threshold of α = 0.05 and was additionally confirmed as significant in multivariable analysis (Table 4, P = 0.002). ^†^Baseline PED area, length, and width survive Bonferroni correction for seven simultaneous tests (adjusted threshold α = 0.0071; all three P < 0.001). Baseline BCVA also achieves corrected significance (adjusted P = 0.032 vs. conventional α = 0.05 threshold) and was confirmed as an independent predictor in multivariable analysis (Table 4, P = 0.010).

### Factors associated with PED area reduction

**Figure 5:**
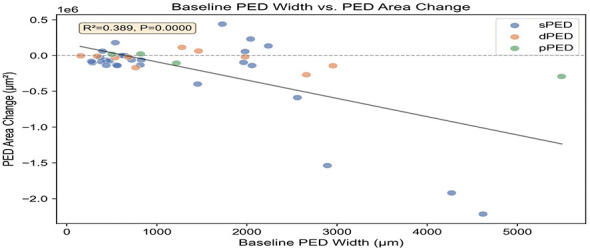
Association between baseline PED width and PED area reduction at 3-month follow-up. Scatter plot of baseline PED width (*x*-axis, μm) against PED area change at 3 months (*y*-axis, μm^2^; post–pre) for all 42 patients. Data points are color-coded by PED subtype as in Figure 4. The regression line with 95% confidence band is from the univariate model (β = −255.24; 95% CI, −358.81 to −151.67; *P* < 0.001; *R*^2^ = 0.389): eyes with wider lesions at baseline showed greater absolute area reductions after SMLT. A negative PED area change indicates lesion reduction. Note that this bivariate relationship is partly tautological: larger baseline lesions can shrink by larger absolute amounts, so the association reflects mathematical coupling and regression toward the mean in addition to any genuine biological determinant of responsiveness. In a sensitivity analysis using proportional area change as the outcome, the association with baseline PED width was substantially attenuated (Supplementary Table S6A). In the multivariable model (Table 4, model C), baseline PED width remained an independent predictor after adjusting for baseline BCVA (*P* < 0.001). OLS, ordinary least squares.

Univariate regression analyses for PED area change identified baseline PED dimensions as the strongest predictors (Table 3). Baseline PED area was the single strongest predictor of PED area reduction (β = −0.496; 95% CI, −0.591 to −0.402; *P* < 0.001; *R*^2^ = 0.738). Baseline PED length (*P* < 0.001; *R*^2^ = 0.514) and PED width (*P* < 0.001; *R*^2^ = 0.389) were also significantly associated with area reduction (Figure 5). Notably, baseline BCVA was significantly associated with PED area change (β = −627,865; 95% CI, −1,036,825 to −218,904; *P* = 0.004; *R*^2^ = 0.194), suggesting that eyes with worse baseline acuity experienced greater anatomic improvement.

These associations are partly explained by mathematical coupling: a lesion with a larger baseline area has more capacity to shrink in absolute terms. In a sensitivity analysis using proportional area change as the outcome, the association with baseline PED area was substantially attenuated (β = −0.0; *P* = 0.523; *R*^2^ = 0.0105). An ANCOVA of posttreatment PED area on baseline PED area (Supplementary Table S6) yielded a regression slope significantly below 1.0 (β = 0.5036; 95% CI, 0.4092 to 0.5981; test of β = 1: *P* = < 0.001), indicating approximately 49.6% regression-to–the-mean effect. Therefore, the strong univariate associations between baseline lesion dimensions and absolute area reduction should not be interpreted as mechanistic predictors of treatment response.

### Multivariable analyses

In a multivariable linear regression model incorporating age, baseline BCVA, ICGA classification, and PED subtype, only baseline BCVA remained a significant independent predictor of BCVA improvement (β = −0.293; 95% CI, −0.467 to −0.118; *P* = 0.002), with the model explaining 25.5% of the variance in BCVA change (adjusted *R*^2^ = 0.255; *F* = 3.336; *P* = 0.011; Table 4). Age (*P* = 0.120), ICGA classification (*P* > 0.25 for both categories), and PED type (*P* > 0.26 for both dPED and pPED relative to sPED) were not independently associated with visual outcomes. A regression diagnostic analysis indicated adequate residual normality (Shapiro–Wilk *P* = 0.124) and exhibited no evidence of heteroscedasticity (Breusch–Pagan *P* = 0.120), although elevated variance inflation factors for ICGA polypoidal (VIF = 6.45) and PED subtype pPED (VIF = 5.06) suggested structural collinearity between these classifications.

**Table 4:** Multivariable linear regression models for BCVA and PED area change.

Variables	*P*	β (95%CI)
Model A: full model^*^	0.011	
Age	0.120	0.01 (−0.00, 0.01)
BCVA (logMAR) at baseline	0.002	−0.29 (−0.47, −0.12)
ICGA focal hyperfluorescence	0.252	−0.11 (−0.31, 0.08)
ICGA polypoidal lesions	0.925	0.02 (−0.48, 0.52)
Drusenoid PED	0.770	0.03 (−0.16, 0.21)
Polypoidal PED	0.259	−0.23 (−0.63, 0.17)
Model B: simple model (BCVA)	0.002	
Age	0.015	0.01 (−0.01, 0.01)
BCVA (logMAR) at baseline	0.001	−0.30 (−0.47, −0.13)
Model C: model with PED width		
BCVA (logMAR) at baseline	0.010	−480,482.86 (−838,060.51, −122,905.21)
PED width at baseline	< 0.001	−219.73 (−319.37, −120.08)

Model A: predictors were age, baseline BCVA, ICGA classification (focal hyperfluorescence and polypoidal lesions vs. vascular dilation [reference]), and PED subtype (dPED and pPED vs. sPED [reference]); N = 42. Model B: predictors restricted to age and baseline BCVA to reduce collinearity and overfitting risk given the sample size of 42; N = 42. Model C: predictors were baseline BCVA and baseline PED width, selected on the basis of clinical plausibility and univariate significance (Table 3); N = 41 (one patient excluded owing to a missing baseline PED width value). For Models A and B, residual normality (Shapiro-Wilk) and homoscedasticity (Breusch–Pagan) assumptions were satisfied. Model C exhibited significant heteroscedasticity (Breusch–Pagan P < 0.001) and non-normal residuals (Shapiro–Wilk P = 0.039), attributable to the right-skewed distribution of baseline PED area; the direction of both predictors is preserved across robust analyses, although conventional significance for baseline BCVA was not maintained under all methods (HC3 P = 0.068; leave-one-out P = 0.054), but exact coefficient magnitudes should be interpreted with caution. CI, 95% confidence interval; OLS, ordinary least squares; μm. micrometers. ^*^Sensitivity analysis excluding one patient with dPED with incomplete registration data (N = 41) yielded materially identical results for Models A and B (baseline BCVA P = 0.003 and P = 0.002, respectively; data not shown).

For PED area change, a multivariable model including baseline BCVA and baseline PED width demonstrated that both variables were independently and significantly associated with the degree of PED area reduction (baseline BCVA: β = −480,483; *P* = 0.010; baseline PED width: β = −220; *P* < 0.001; adjusted *R*^2^ = 0.462; Table 4). However, this model showed evidence of heteroscedasticity (Breusch-Pagan *P* < 0.001) and non-normal residuals (Shapiro–Wilk *P* = 0.039), and the point estimates should therefore be interpreted with caution.

### Subgroup analysis: patients with sPED

Given that sPED represented the largest subgroup and was the only group to demonstrate statistically significant PED area reduction, we performed a dedicated subgroup analysis of the 27 patients with sPED (Table 5). Baseline BCVA was the only significant predictor of BCVA improvement in this subgroup (β = −0.278; 95% CI, −0.515 to −0.041; *P* = 0.024; *R*^2^ = 0.189). Neither the number of laser spots (*P* = 0.189), baseline PED width (*P* = 0.658), baseline PED area (*P* = 0.875), nor the magnitude of PED area change (*P* = 0.740) was significantly associated with visual outcomes in the sPED subgroup.

**Table 5:** Univariate linear regression analysis of factors associated with BCVA change in the serous PED subgroup (*N* = 27).

Variable	*P*	β (95%CI)
Age	0.627	0.00 (−0.01, 0.01)
Gender	0.647	0.04 (−0.14, −0.14)
BCVA (LogMAR) at baseline	0.024	−0.28 (−0.51, −0.04)
PED length at baseline	0.293	−0.00 (−0.00, 0.00)
PED width at baseline	0.658	0.00 (−0.00, 0.00)
PED area at baseline	0.875	0.00 (−0.00, 0.00)
Laser spots	0.189	0.00 (−0.00, 0.00)
PED area change	0.740	0.00 (−0.00, 0.00)

Analyses were restricted to the 27 patients with sPED, who represented the only subgroup to demonstrate statistically significant PED area reduction following SMLT. Each predictor was evaluated in a separate simple linear regression model. PED area change (post–pre, μm^2^) was included as a predictor to test for a correlation between anatomic and visual improvement. No predictor other than baseline BCVA reached statistical significance.

## Discussion

**Figure 6:**
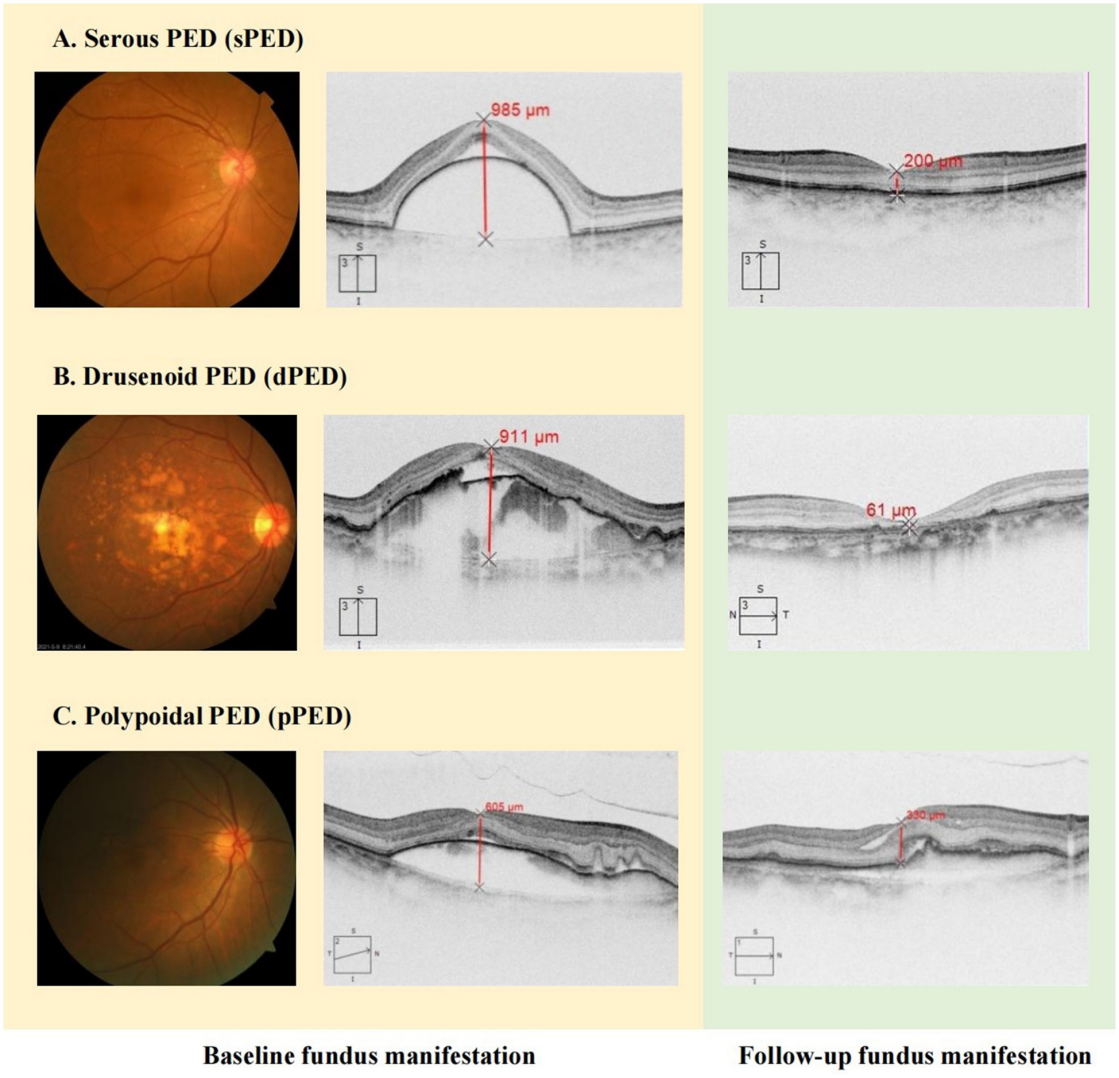
Representative imaging of each pigment epithelium detachment subtype at baseline and 3-month follow-up. Representative color fundus photographs and optical coherence tomography (OCT) B-scan images at baseline, and B-scan images at 3-month follow-up were presented.

This study analyzed the basic characteristics, ocular features, treatment conditions, and follow-up data of 42 patients with different types of PED (Figure 6). Because this was an uncontrolled retrospective case series, all observed pre–post changes should be interpreted as associations rather than treatment effects of SMLT. PED area decreased from baseline to 3 months in all three subtypes, with the only statistically significant reduction observed in the sPED subgroup (*P* = 0.043; confirmed by the Wilcoxon signed-rank test, *P* = 0.015). Visual acuity improvements were modest overall (mean change, −0.07 LogMAR) and did not reach statistical significance within individual PED type groups, although a trend toward improvement was observed in the sPED subgroup (*P* = 0.079; Cohen's *d* = −0.35). Baseline BCVA was the strongest predictor of visual outcome and an independent predictor of anatomic outcome in the multivariable analysis; however, its association with anatomic outcome did not retain conventional significance under all robust sensitivity analyses.

**Figure 7:**
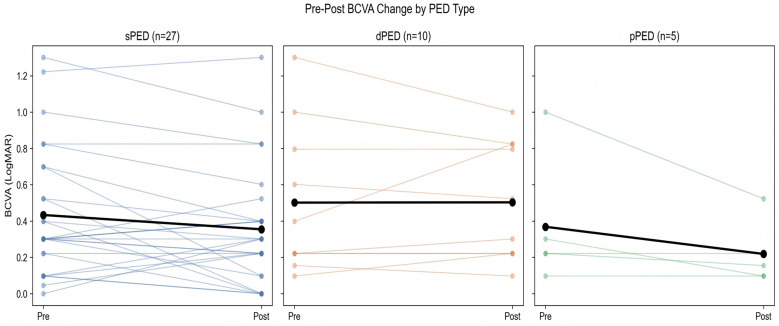
Individual pretreatment and posttreatment BCVA by PED subtype. Paired line plot showing individual BCVA values (LogMAR) at baseline and at 3-month follow-up for each of the 42 patients, faceted by PED subtype. Each line connects one patient's pretreatment **(left)** and posttreatment **(right)** value. Lines sloping downward indicate improvement; upward-sloping lines indicate deterioration. Bold lines with filled markers represent group means at each time point. The plot illustrates the substantial interpatient variability in visual response within each subtype and the absence of a uniform directional shift in the dPED group.

Upon reviewing the patients, we found that sPED was primarily diagnosed as CSC, dPED as drusenoid PED, and pPED as polypoidal choroidal vasculopathy (PCV). Correspondingly, patients with sPED were more numerous, younger (mean age 53.00 vs. 77.20 years for dPED and 71.00 years for pPED; *P* < 0.001), and had the largest observed reductions in BCVA and PED area (Figure 7), although these findings should not be interpreted as treatment effects in the absence of a control group. The significant gender imbalance among groups—with all five patients with pPED being male—is consistent with the known male predominance of PCV (12, 13). The finding that baseline BCVA was the only significant predictor of visual improvement suggests that early treatment, particularly when visual acuity is relatively well preserved, may provide greater benefit to patients. However, approximately 29% of this association may be attributable to regression to the mean, and this finding should therefore be interpreted with caution.

Our findings raise the possibility that SMLT could be explored as an adjunctive option for pPED, although a controlled comparison is required before any clinical recommendation. The recommended treatments for pPED primarily involve anti-VEGF therapy, photodynamic therapy (PDT), or a combination of both (12, 13, 16). However, the high cost of PDT and anti-VEGF treatments has become a significant financial burden for patients, leading to delays in treatment initiation. The scarcity of photosensitizers in China, which are essential for PDT, further limits the application of this therapy (14, 15, 18). Although our study indicates that the majority of patients with pPED still required anti-VEGF therapy after SMLT (60.0%), the PED area decreased in these patients during follow-up. Given the lower cost of SMLT, it may warrant evaluation as an early intervention or adjunctive therapy for pPED or early-stage PCV in prospective controlled studies, which may provide a new option in the management of PCV. This finding warrants further investigation in larger, controlled studies.

Between-group comparisons of change scores should be interpreted as exploratory, given subgroup sizes as small as five and unequal variances; sensitivity analyses using Welch's ANOVA, Kruskal–Wallis, and permutation-based tests yielded concordant non-significant findings.

This study has several limitations. First, because the micropulse laser is a relatively new treatment method and not a first-line therapy for PED, the study included a limited sample size of 42 patients (42 eyes), with only 10 patients with dPED and 5 patients with pPED. This limited sample size resulted in low statistical power for subgroup analyses, as reflected by wide confidence intervals for effect sizes (e.g., pPED Cohen's *d* = −0.74; 95% CI, −2.07 to 0.58). The small sample size also precluded separate factor analyses for the dPED and pPED groups. Second, due to the lack of photosensitizer availability, we did not conduct a comparative analysis of the therapeutic outcomes of SMLT on pPED with those of PDT. Third, the observational regression-to-the-mean effect (approximately 29% of the baseline BCVA–outcome association) limits causal interpretation of the BCVA findings. Fourth, the multivariable model for PED area change exhibited heteroscedasticity, non-normal residuals, and one highly influential observation. Although the direction of the two independent predictors was preserved under HC3 robust standard errors, BCa bootstrap confidence intervals, and a leave-one-out sensitivity analysis—note that conventional significance for baseline BCVA was not fully preserved across all robust analyses: HC3 *P* = 0.068, leave-one-out *P* = 0.054—the exact coefficient magnitudes should be interpreted with caution, and the model should be regarded as descriptive rather than predictive. Finally, the 3-month follow-up period may be insufficient to capture the full treatment effect, particularly for dPED and pPED. A sensitivity analysis excluding one patient with incomplete registration data (*N* = 41) yielded materially identical results, supporting the robustness of the primary findings. Further investigation in larger cohorts is needed to determine whether SMLT can achieve clinically meaningful reductions in PED dimensions and BCVA gains in dPED and pPED eyes.

## Data Availability

The original contributions presented in the study are included in the article/Supplementary material, further inquiries can be directed to the corresponding author.
