# Supplementary material for: Subthreshold micropulse laser for retinal pigment epithelium detachment: subtype-stratified outcomes and predictors of treatment response
Source: Front Med (Lausanne). 2026 Jun 22;13:1810012. doi: 10.3389/fmed.2026.1810012 (PMC13333342; doi:10.3389/fmed.2026.1810012)
Supplement: Supplementary file 1 [file Table_1.docx]

**1. SUPPLEMENTARY TABLES**

**Supplementary Table S1. Sensitivity Analysis: Key Outcomes and Regression Results Excluding One Patient with Incomplete Registration Data (N=41)**

| **Outcome** | **Primary (N=42)** | **Sensitivity (N=41)** |
| --- | --- | --- |
| Mean age, years | 60.90 ± 16.08 | 60.66 ± 16.22 |
| Male, n (%) | 25 (59.5%) | 24 (58.5%) |
| Baseline BCVA, LogMAR | 0.44 ± 0.36 | 0.43 ± 0.36 |
| BCVA change, β (BCVA baseline) | −0.293 (P=0.002) | −0.290 (P=0.003) |
| PED area change, β (PED width) | −219.7 (P<0.001) | −217.5 (P<0.001) |
| Full model Adj. R² | 0.255 | 0.258 |

One drusenoid PED patient (age 65 years, female) was included in the primary analysis (N=42) despite the absence of a sequence number (na01) or patient identification number in the registry. To assess whether this inclusion materially influenced the findings, all primary analyses were repeated with this patient excluded (N=41: 27 sPED, 9 dPED, 5 pPED). Columns compare the primary N=42 result and the N=41 sensitivity result. No finding changed materially in direction, magnitude, or statistical significance category. The primary N=42 analysis is therefore considered robust.

**Supplementary Table S2. Regression-to-the-Mean Analysis: ANCOVA of Post-Treatment BCVA on Baseline BCVA**

| **Model** | **β (95% CI)** | **P-value** | **R²** |
| --- | --- | --- | --- |
| Model I: baseline BCVA only | 0.712 (0.545, 0.879) | <0.001 | 0.650 |
| Model I: test of β=1.0 | t=−3.485 | P=0.001 | ≈29% RTM |
| Model II: + dPED | β=+0.101 (n.s.) | 0.150 | — |
| Model II: + pPED | β=−0.091 (n.s.) | 0.322 | — |
| Model II: baseline BCVA | β=0.708 (0.533, 0.883) | <0.001 | 0.662 |

To quantify the contribution of regression to the mean to the observed association between baseline BCVA and visual improvement, an ANCOVA-style model was fitted with post-treatment BCVA (LogMAR) as the dependent variable and baseline BCVA (LogMAR) as the sole predictor (Model I) and additionally adjusted for PED type (Model II). A slope (β) significantly less than 1.0 indicates that the post-treatment value does not fully track the baseline value, implying partial regression toward the mean. Model I: β=0.712 (95% CI, 0.545 to 0.879; P<0.001; R²=0.650); the test of β=1.0 was significant (t=−3.485; P=0.001), indicating approximately 29% regression to the mean. After adjustment for PED type (Model II), neither dPED (β=+0.101; P=0.150) nor pPED (β=−0.091; P=0.322) was a significant predictor of post-treatment BCVA, consistent with PED type not being an independent determinant of visual outcome.

**Supplementary Table S3. Normality Testing of Primary Outcome Variables (Shapiro-Wilk Test)**

| **Variable** | **Group** | **W Statistic** | **P-value** |
| --- | --- | --- | --- |
| BCVA change | Overall (N=42) | 0.956 | 0.135 |
| BCVA change | sPED (n=27) | 0.960 | 0.359 |
| BCVA change | dPED (n=10) | 0.948 | 0.631 |
| BCVA change | pPED (n=5) | 0.882 | 0.332 |
| PED area change | Overall (N=42) | 0.581 | <0.001 |
| PED area change | sPED (n=27) | 0.648 | <0.001 |
| PED area change | dPED (n=10) | 0.929 | 0.442 |
| PED area change | pPED (n=5) | 0.793 | 0.071 |

Shapiro-Wilk tests were performed to assess the distributional assumptions underlying parametric analyses. BCVA change satisfied normality in all subgroups and overall (all P>0.13), supporting use of paired t-tests and ANOVA. PED area change was severely non-normally distributed in the sPED group (W=0.648, P<0.001) and overall (W=0.581, P<0.001), driven by a right-skewed distribution with a minority of patients experiencing very large area reductions. This motivated the use of Wilcoxon signed-rank tests as confirmatory analyses for PED area change. Importantly, non-parametric testing strengthened the sPED finding (Wilcoxon P=0.015 vs. paired t P=0.043), and all other comparisons yielded consistent conclusions across parametric and non-parametric approaches.

**Supplementary Table S4. Multivariable Regression Diagnostic Summary for Models A, B, and C (Table 5)**

| **Diagnostic** | **Model** | **Result** |
| --- | --- | --- |
| Residual normality (S-W) | Model A (full BCVA) | W=0.958, P=0.124 ✓ |
| Residual normality (S-W) | Model B (simple BCVA) | W=0.972, P=0.382 ✓ |
| Residual normality (S-W) | Model C (PED area) | W=0.893, P=0.039 ✗ |
| Heteroscedasticity (B-P) | Model A | P=0.120 ✓ |
| Heteroscedasticity (B-P) | Model B | P=0.013 ⚠ |
| Heteroscedasticity (B-P) | Model C | P<0.001 ✗ |
| Collinearity (max VIF) | Model A | VIF=6.45 (ICGA polypoidal) |
| Influential obs (Cook's D) | Model C | Cook's D=0.90 (1 obs) |

Regression diagnostics were computed for each multivariable model as follows: (1) Residual normality: Shapiro-Wilk test applied to model residuals. (2) Heteroscedasticity: Breusch-Pagan test (χ² statistic and P-value). (3) Collinearity: variance inflation factors (VIF) for each predictor. (4) Influential observations: Cook's distance, with threshold set at 4/N. Model A (full BCVA model, N=42): residuals were normally distributed (W=0.958, P=0.124), no significant heteroscedasticity (Breusch-Pagan P=0.120); elevated VIF for ICGA polypoidal (6.45) and pPED type (5.06) reflects structural collinearity between these classifications (all polypoidal ICGA patients had pPED). Model B (simplified BCVA model, N=42): residuals were normally distributed; mild heteroscedasticity detected (Breusch-Pagan P=0.013). Model C (PED area model, N=41): significant heteroscedasticity (Breusch-Pagan P<0.001) and non-normal residuals (Shapiro-Wilk P=0.039); one highly influential observation (Cook's D=0.90); condition number 5,938 (scale-driven). These findings are discussed in the Limitations section of the main text.

**Supplementary Table S5. Multiple Comparison Analysis: Bonferroni-Corrected P-Values for Univariate Regression Tests**

| **Predictor** | **Raw P-value** | **Bonferroni P** | **Significant?** |
| --- | --- | --- | --- |
| **BCVA Change (9 tests, α=0.0056)** |  |  |  |
| Age | 0.352 | 1.000 | NS |
| Gender | 0.305 | 1.000 | NS |
| Baseline BCVA (LogMAR) | 0.001 | 0.011 | Yes |
| Baseline PED length | 0.274 | 1.000 | NS |
| Baseline PED width | 0.254 | 1.000 | NS |
| Baseline PED area | 0.471 | 1.000 | NS |
| Laser spots | 0.721 | 1.000 | NS |
| ICGA focal hyperfluorescence | 0.636 | 1.000 | NS |
| ICGA polypoidal lesions | 0.822 | 1.000 | NS |
| **PED Area Change (9 tests, α=0.0056)** |  |  |  |
| Age | 0.502 | 1.000 | NS |
| Gender | 0.596 | 1.000 | NS |
| Baseline BCVA (LogMAR) | 0.004 | 0.036 | Yes |
| Baseline PED length | 0.000 | <0.001 | Yes |
| Baseline PED width | 0.000 | <0.001 | Yes |
| Baseline PED area | 0.000 | <0.001 | Yes |
| Laser spots | 0.968 | 1.000 | NS |
| ICGA focal hyperfluorescence | 0.479 | 1.000 | NS |
| ICGA polypoidal lesions | 0.564 | 1.000 | NS |

Nine univariate regression models were fitted per primary outcome variable (BCVA change and PED area change), yielding a Bonferroni-corrected significance threshold of α=0.05/9=0.0056. For BCVA change, baseline BCVA (raw P=0.001; Bonferroni-adjusted P=0.011) did not survive the strict Bonferroni threshold but was confirmed as significant in multivariable analysis (Table 5, P=0.002), which inherently adjusts for multiple covariates and provides stronger evidence than corrected univariate tests. For PED area change, the three baseline PED dimension predictors (area, length, width) all survived Bonferroni correction (all adjusted P<0.001). No predictor other than baseline BCVA achieved raw P<0.05 for BCVA change; no predictor other than baseline PED dimensions and baseline BCVA achieved raw P<0.05 for PED area change.

**Supplementary Table S6. Sensitivity Analyses for the Baseline-Lesion-Size → PED Area Change Associations: Proportional Change Outcome and ANCOVA Quantifying Regression Towards the Mean**

1. Univariate regressions with proportional PED area change [(post − pre)/pre × 100%] as the outcome

| **Predictor** | **β** | **95% CI** | **P** | **R²** |
| --- | --- | --- | --- | --- |
| Baseline PED area | -0.0 | -0.0 to 0.0 | 0.523 | 0.0105 |
| Baseline PED length | -0.0012 | -0.0029 to 0.0004 | 0.139 | 0.0554 |
| Baseline PED width | -0.0001 | -0.0003 to 0.0002 | 0.673 | 0.0046 |
| Baseline BCVA (LogMAR) | -0.6367 | -1.569 to 0.2956 | 0.175 | 0.0466 |
| Age | 0.0051 | -0.0155 to 0.0257 | 0.618 | 0.0064 |

1. ANCOVA of post-treatment PED area on baseline PED area

| **Model** | **β** | **95% CI** | **P** | **R² / Adj. R²** |
| --- | --- | --- | --- | --- |
| Model I — Post-area ~ Baseline-area | 0.5036 | 0.4092 to 0.5981 | <0.001 | R²=0.7438 |
| Test of slope=1 | t=-10.6207 |  | <0.001 | RTM≈49.6% |
| Model II — Post-area ~ Baseline-area + PED type |  |  |  | Adj. R²=0.745 |
| Baseline area | 0.5081 |  | <0.001 |  |
| dPED | 147555.8622 |  | 0.136 |  |
| pPED | 152749.6645 |  | 0.237 |  |

This table accompanies the sensitivity analyses described in the revised Results (Factors Associated with PED Area Reduction). Panel A demonstrates that the strong univariate associations between baseline PED dimensions and absolute area reduction (Table 3; R² up to 0.738) are substantially attenuated when proportional change is used as the outcome, indicating that the absolute-change associations are inflated by mathematical coupling (larger lesions have more room to shrink in absolute terms). Panel B presents an ANCOVA of post-treatment PED area on baseline PED area; a regression slope significantly less than 1.0 indicates regression toward the mean, analogous to Supplementary Table S2 for the BCVA outcome.

Abbreviations: RTM = regression toward the mean.

**2. SUPPLEMENTARY Figure**

**Supplementary Figrue S1. Regression Diagnostic Plots for Multivariable Models A, B, and C**

**
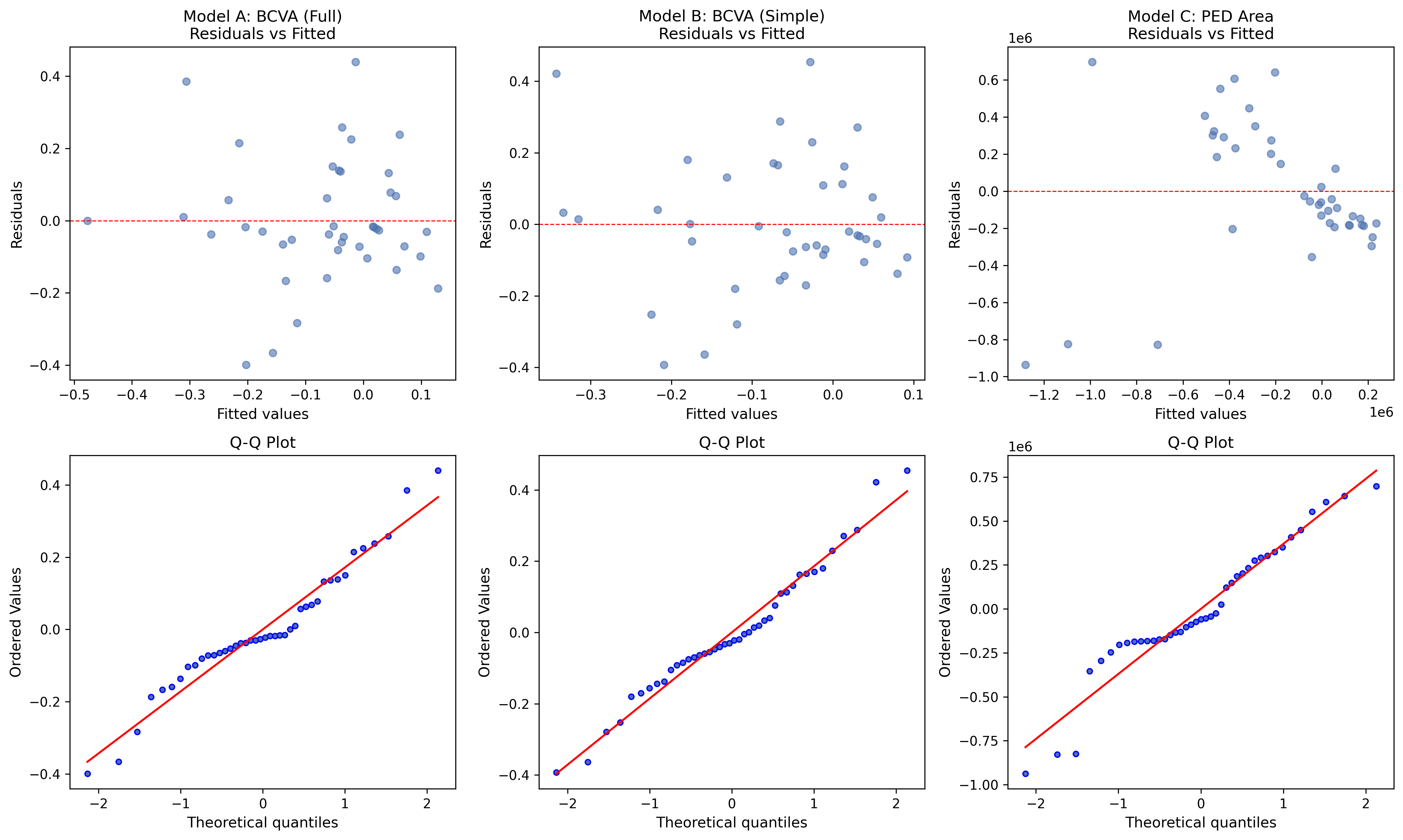
**

Four standard diagnostic plots are displayed for each multivariable model (Models A, B, C; described in Table 5 and Supplementary Table S4). For each model, panels show: (A) Residuals vs. Fitted values — used to assess linearity and identify heteroscedasticity; (B) Normal Q-Q plot of standardised residuals — used to assess normality of residuals; (C) Scale-Location plot (√|standardised residuals| vs. fitted values) — used to confirm homoscedasticity; (D) Residuals vs. Leverage plot with Cook's distance contours — used to identify influential observations. Models A and B show adequate fit. Model C (PED area) demonstrates a clear fan-shaped pattern in the Residuals vs. Fitted plot consistent with heteroscedasticity (Breusch-Pagan P<0.001), and the Q-Q plot shows deviation in the right tail consistent with positive skewness of PED area change.

Abbreviations: OLS = ordinary least squares.

**3. MASTER LIST OF ABBREVIATIONS**

The following abbreviations are used throughout the tables, figures, and supplementary materials:

| **Abbreviation** | **Definition** |
| --- | --- |
| ANCOVA | analysis of covariance |
| ANOVA | analysis of variance |
| BCVA | best-corrected visual acuity |
| β | unstandardised regression coefficient |
| CI | 95% confidence interval |
| Cohen's d | standardised mean difference effect size |
| CSC | central serous chorioretinopathy |
| dPED | drusenoid pigment epithelium detachment |
| FFA | fluorescein fundus angiography |
| ICGA | indocyanine green angiography |
| IQR | interquartile range |
| LogMAR | logarithm of the minimum angle of resolution |
| mW | milliwatts |
| NS | not significant |
| OCT | optical coherence tomography |
| OLS | ordinary least squares |
| PCV | polypoidal choroidal vasculopathy |
| PED | pigment epithelium detachment |
| pPED | polypoidal pigment epithelium detachment |
| PRN | pro re nata (as needed) |
| R² | coefficient of determination |
| RPE | retinal pigment epithelium |
| SD | standard deviation |
| SMLT | subthreshold micropulse laser treatment |
| sPED | serous pigment epithelium detachment |
| VIF | variance inflation factor |
| VEGF | vascular endothelial growth factor |
| μm | micrometres |
| μm² | square micrometres |
| W | Shapiro-Wilk test statistic |
